# Cartilage disruption patterns associated with percutaneous intramedullary screw fixation for metatarsal fractures: a cadaveric study

**DOI:** 10.1007/s00402-026-06457-3

**Published:** 2026-09-02

**Authors:** Bailey Keith, Joseph Burke, Kelly Hynes, James Baker, Jeannie Huh

**Affiliations:** 1 Department of Orthopaedic Surgery, San Antonio Military Medical Center, San Antonio, USA; 2 Department of Orthopaedic Surgery, Washington University in St. Louis, St Louis, USA

**Keywords:** Metatarsal, Articular Cartilage, Intramedullary Screw Fixation, Percutaneous Fixation, Metatarsal Fractures

## Abstract

**Purpose:**

Intramedullary screw fixation is an emerging technique for operative metatarsal fractures, yet the extent and location of articular cartilage disruption from retrograde screw insertion remain undefined. This study quantifies cartilage surface disruption and maps injury locations in the metatarsal head following screw placement.

**Methods:**

Fourteen fresh-frozen cadaveric feet (56 lesser metatarsals) underwent standardized retrograde intramedullary screw fixation using 3.6 mm screws. Following dissection, the total articular surface area and cartilage defect were measured to calculate the percentage of disruption. The cartilage surface was divided into quadrants (dorsal-to-plantar) and thirds (medial-to-lateral) to determine screw trajectory.

**Results:**

Mean cartilage disruption increased progressively from the 2nd to 5th metatarsals: 10.17%, 11.2%, 14.93%, and 17.43%, respectively. All screws were located within the middle third in the medial-to-lateral plane. In the dorsal-to-plantar plane, the majority of screws were positioned in the 2nd and 3rd quadrants. No screws were placed in the medial or lateral thirds.

**Conclusion:**

Retrograde intramedullary screw fixation results in measurable cartilage disruption, with a tendency for screw placement in the middle third in the medial-to-lateral plane and 2nd and 3rd dorsal-to-plantar quadrants. While the relative cartilage surface disruption ranges from approximately 10% to 17%, increasing in more lateral metatarsals, the clinical implications remain unknown. These findings define the articular disruption of intramedullary screws in metatarsal fractures and highlight the need for future studies on the clinical outcomes after undergoing this fixation construct.

## Introduction

**Fig. 1:**
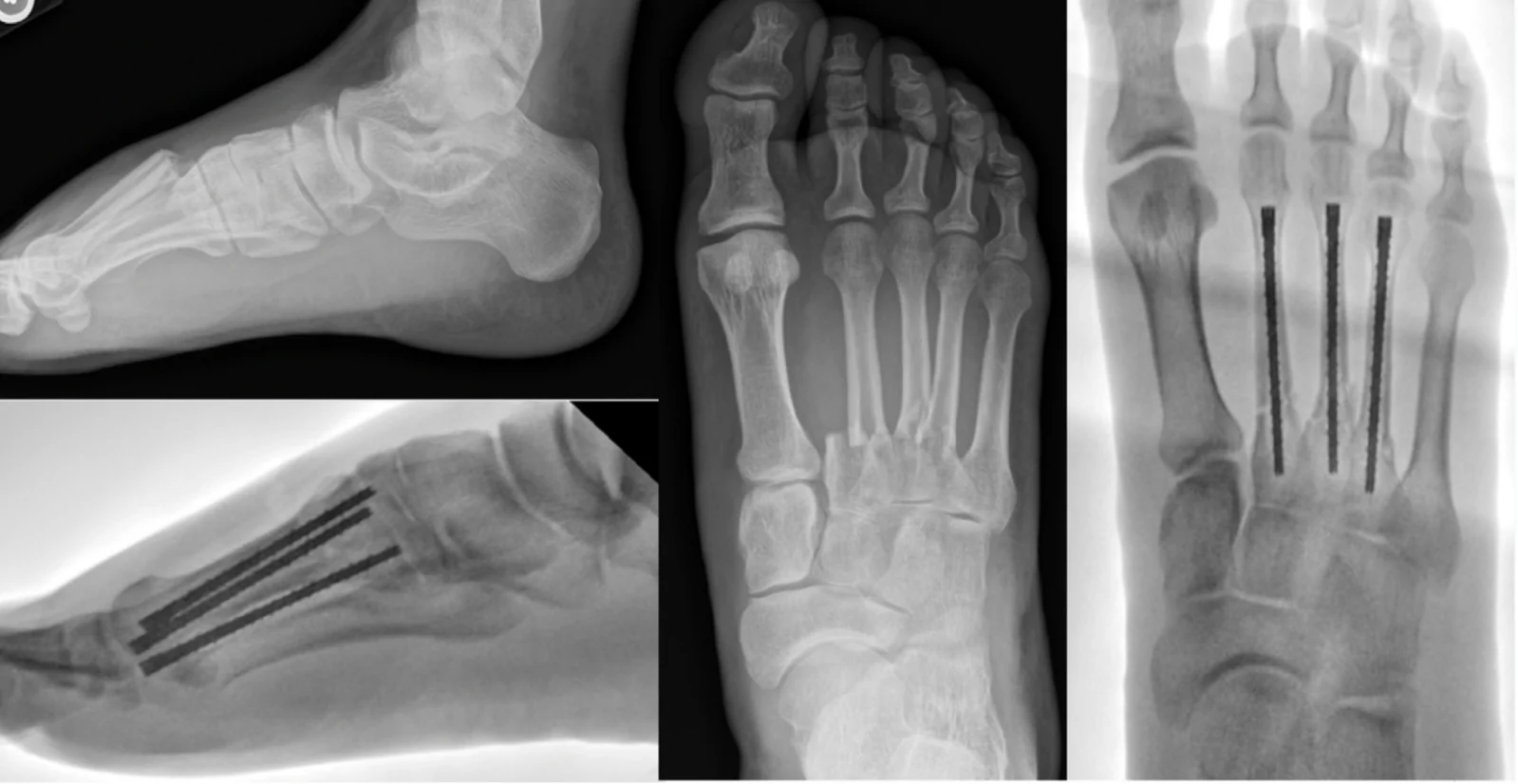
Multiple displaced extra-articular (2nd -4th ) metatarsal fractures treated with intramedullary screw fixation in a 35-year-old polytrauma patient

Metatarsal fractures account for about 35% of all foot fractures and 5–6% of all skeletal injuries [1]. Indications for operative management include significant displacement, open fracture, and multiple metatarsal fractures. Surgical stabilization can be achieved using Kirschner wires (K-wire), screws, or plate fixation, depending on the fracture pattern [1, 2]. K-wires are frequently used for metatarsal fracture fixation due to the ability to insert them percutaneously, and therefore preserve the soft tissue envelope around the fracture. However, they are not without complications. Because the wires are placed temporarily and exposed to the external environment until removed, pin site infections are a significant concern and can negatively impact outcomes by necessitating antibiotics, early removal of the hardware, or even revision surgery if fixation fails [3]. Such issues can lead to increased healthcare costs and delayed return to daily activities for the patient [4]. In light of the potential complications associated with percutaneous K-wire fixation of metatarsal fractures, a more permanent, yet still percutaneous, fixation option using intramedullary screws has emerged as a favorable alternative, offering stable compression at the fracture site, early range of motion, and minimal soft tissue disruption (Fig. 1) [5]. 

Intramedullary screw fixation is increasingly being used for the management of unstable phalangeal and metacarpal fractures in the upper extremity [3]. Multiple studies have reported low complication rates, high union rates, and excellent postoperative range of motion associated with this technique [3, 6]. One potential area of concern with intramedullary screw fixation is the risk of damage to articular cartilage [5, 7]. Cartilage injury location and extent has been studied in the hand as it may contribute to pain during motion or predispose to early arthritis [6]. Similar investigations have not been conducted in the lower extremity, despite these screws existing as a fixation option for metatarsal fractures.

When used for metatarsal fractures, intramedullary screws are typically inserted via a plantar approach and retrograde technique, inevitably violating the metatarsal head cartilage. The degree of cartilage damage has not been defined. Understanding the amount and location of the cartilage defect could provide insight on the likelihood of resultant arthritis. Therefore, the purpose of this study was to quantify the percentage of cartilage disruption and determine the relative location within the metatarsal head with insertion of these intramedullary screws.

## Methods

This study was approved by the Institutional Review Board at our institution. Seven pairs of fresh-frozen cadaveric feet were procured. All lesser metatarsals underwent intramedullary screw fixation and were evaluated for a total of 56 metatarsals in 14 feet. No formal power analysis was performed in this study as this was a cadaveric descriptive study with a primary outcome of proportion of metatarsal head cartilage injury and a secondary outcome of injury location. Each cadaveric foot was given a numeric label, and the feet were designated as right or left for record keeping, i.e. 1R, 1 L, 2R, 2 L, etc. Extremities were inspected to ensure no obvious deformities or prior surgeries were present.

**Fig. 2:**
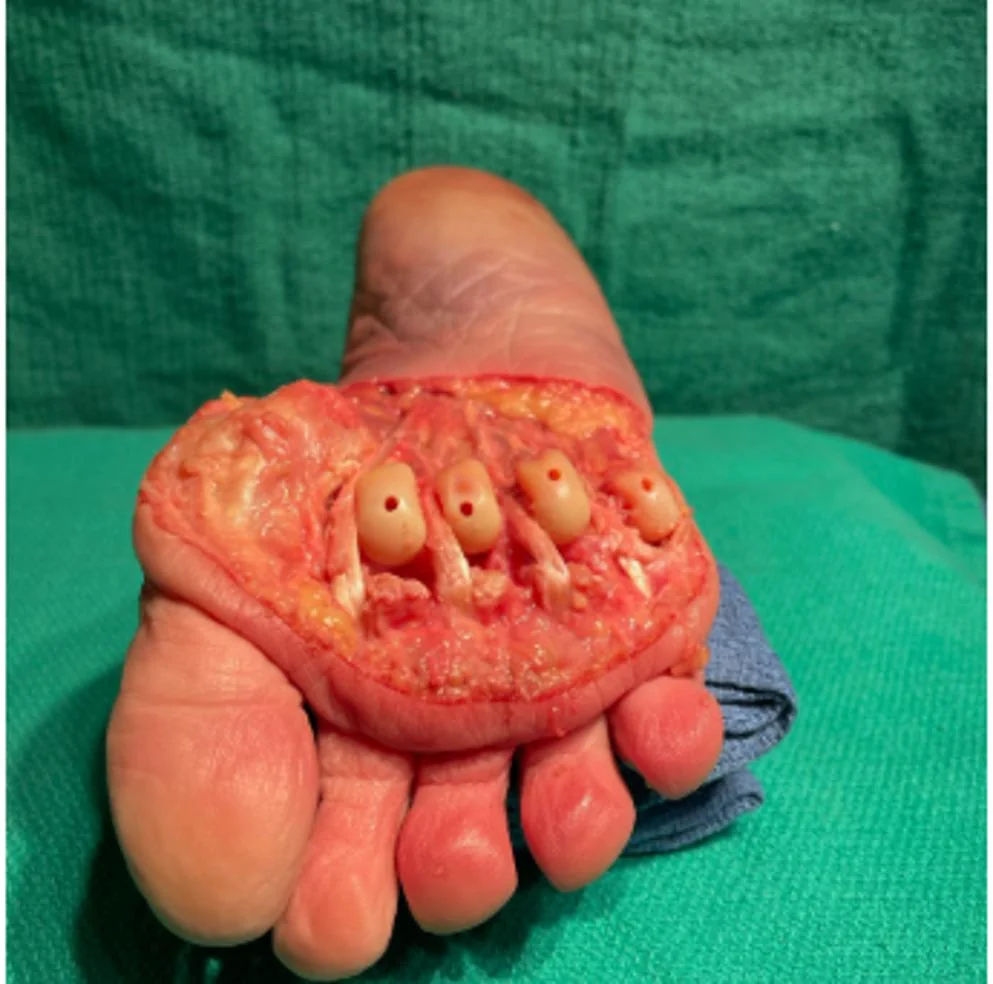
Plantar dissection exposing the location of the drill holes

For each specimen, retrograde intramedullary screw fixation of the lesser metatarsals was performed according to the manufacturer’s implant technique guide (Exosmed Innate System, Accumed, Hillsboro, OR). While holding each toe in maximal dorsiflexion at the metatarsophalangeal joint (MTP) joint, a 0.045 inch guidewire was percutaneously introduced from the plantar aspect of the foot on the metatarsal head. The starting point was determined under fluoroscopy and corresponded to the center of the metatarsal head on the anteroposterior view and matched the trajectory of the intramedullary canal on the lateral view. The wire was then advanced in retrograde fashion along the central axis of the metatarsal canal to the subchondral base. A 5 mm (mm) vertical stab incision was made with a #15 blade along the wire, passing through the skin and subcutaneous tissue. A small straight mosquito was then used to carefully spread the deep tissues in line with the stab incision down to the metatarsal head. This technique facilitated reaming and implant placement while minimizing disruption to the soft tissues. Next, a cannulated 2.9 mm reamer was advanced over the wire. Reaming was completed without a soft tissue protector to replicate the manufacturer’s technique. Finally, a cannulated 3.6 mm fully threaded screw (ExosMed Innate System, Acumed, Hillsboro, OR) was inserted over the wire until the screw was subchondral and buried beneath the articular surface of the metatarsal head. The 3.6 mm screw was utilized for all metatarsals to minimize variability with reamer size. This process was completed for each metatarsal. Once implantation was complete for each specimen, the skin and subcutaneous tissues overlying the metatarsal cartilage were dissected to obtain adequate visualization for measurement of articular disruption (Fig. 2).

Data were collected from each lesser metatarsal following instrumentation and subsequent dissection. The cartilage defect from the screw insertion was identified. The diameter of the screw inserted was 3.6 mm, thus we estimated a defect size of 31.98 mm² (area of 3.6 mm screw). The medial to lateral width of the cartilage was measured, as well as the dorsal to plantar cartilage distance. Measurements were made with a digital caliper (Husky 6 in. digital caliper; accuracy tolerance, ± 0.025 mm), by a single observer. The width and length of the total cartilage was then multiplied to get the total surface area of the articular cartilage. The defect was then divided by the total cartilage area to get a percentage of total cartilage disruption for each metatarsal.

**Fig. 3:**
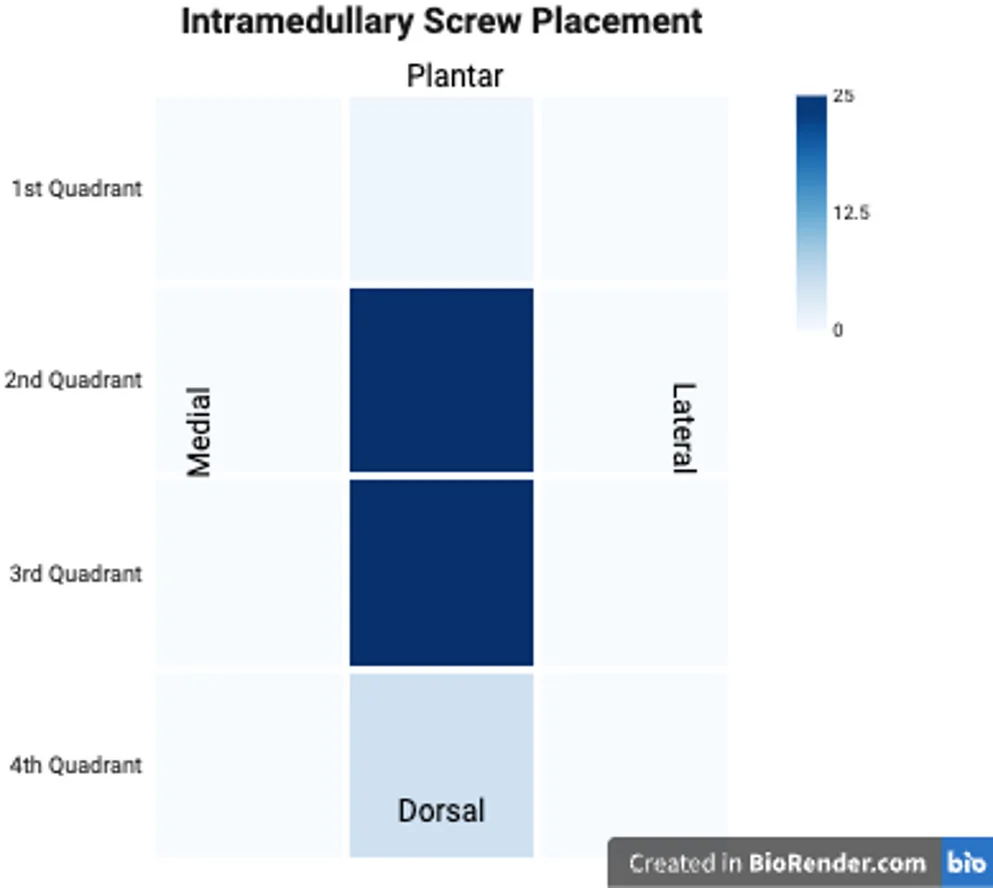
Heat Map of Metatarsal Head Screw Insertion (Cartilage Disruption) Location (Total of 56 Screws, Quadrants Differ Based on Metatarsal Size, defined in Results)

Additionally, we measured the distance from the lowest point (most plantar aspect) of the cartilage to the defect. We then divided the total length of the cartilage from dorsal (top) to plantar (bottom) into four equal sections, creating quadrants. This allowed us to identify the specific quadrant where the defect was located, which helped map the position of the screw inserted into the metatarsal head. For example, quadrant one represented the plantar fourth of the metatarsal cartilage, while quadrant four represented the dorsal quarter of the cartilage surface area. Next, we applied a similar approach in the medial (inner) to lateral (outer) direction by dividing the length into three equal sections, or thirds. For clarity, we created a heat map to illustrate the approximate location of the drill holes on the articular cartilage. **(**Fig. 3**)** For simplicity, all drill holes were categorized as being in the middle third of the medial to lateral plane since no holes were made in the medial or lateral zones. Finally, we pooled this data to calculate the mean values and standard deviations.

## Results

There were 56 intramedullary screws inserted into the metatarsal articular surfaces with mean surface areas (mm^2^) found to be 347.57, 312.07, 239.36, and 202 for the 2nd-5th, respectively **(**Table 1**)**. The mean percentage of cartilage disruption by the head of the screw for the 2nd metatarsal was 10.17% (+/- 3.4), 11.2% (+/- 3.3) for the 3rd, 14.93% (+/- 5.3) for the 4th, and 17.43% (+/- 5.8) for the 5th **(**Table 1**)**. The total articular surface for each of the metatarsals was measured in the dorsal to plantar direction resulting in 30 mm for the 2nd and 3rd metatarsals, 26 mm for the 4th, and 24 mm for the 5th. Each metatarsal surface was divided into 4 quadrants of 7.5 mm for the 2nd and 3rd, 6.5 mm for the 4th, and 6 mm for the 5th relative to the plantar edge of the cartilage. **(**Table 2**)** While all screws were placed in the middle third of the articular surface in the medial-to-lateral plane, in the dorsal-to-plantar plane the largest percentage of screws were placed in the 2nd and 3rd quadrants **(**Table 3**).**

**Table 1:** Mean Surface Area of Metatarsal Head and Percentage of Cartilage Disruption

2nd Metatarsal	Mean Total Articular Surface Area (mm²)	SD	Mean Percentage of Cartilage Disruption (%)	SD
	347.57	113.59	10.17	3.46
3rd Metatarsal	312.07	100.11	11.2	3.3
4th Metatarsal	239.36	79.01	14.93	5.31
5th Metatarsal	202	62.36	17.43	5.81

*MM* millimeters, *SD* Standard Deviation, Area of 3.6 mm diameter screw = 31.98 mm²

**Table 2:** Number of Screws Placed Based on Quadrants and Distance from Plantar Cartilage for Each Metatarsal

Metatarsal	Quadrant	Distance from Plantar Cartilage (mm)	Screws Placed (*n*)	Percentage of Screws (%)
Combined (2nd–3rd)	1st	0–7.5	0	0.00
	2nd	7.5–15	13	46.43
	3rd	15–22.5	15	53.57
	4th	22.5–30	0	0.00
4th Metatarsal	1st	0–6.5	0	0.00
	2nd	6.5–13	5	35.71
	3rd	13–19.5	4	28.57
	4th	19.5–26	5	35.71
5th Metatarsal	1st	0–6	1	7.14
	2nd	6–12	7	50.00
	3rd	12–18	6	42.86
	4th	18–24	0	0.00

If a screw was placed at a transition point between quadrants, the screw will be counted in the quadrant below it. For example, 7.5 mm would be counted in the 1st quadrant.

**Table 3:** Number of Screws Placed Based on Quadrants and Distance from Plantar Cartilage, All 56 Screws Included

Zone	Number of Screws Placed	Percentage of Screws (%)
1st Quadrant	1	1.79
2nd Quadrant	25	44.64
3rd Quadrant	25	44.64
4th Quadrant	5	8.93

## Discussion

This study aimed to quantify the articular cartilage disruption associated with intramedullary screw placement in the lesser metatarsals and define the approximate zone of injury to the articular cartilage on the metatarsal head. Our findings demonstrated a progressive increase in the mean percentage of cartilage disrupted from the 2nd to the 5th metatarsal, with the 5th metatarsal experiencing the greatest relative disruption. This likely reflects the decreasing articular surface area in the more lateral metatarsals, which inherently results in a greater proportion of surface involvement by the screw head. While all screws were placed in the middle third of the articular surface to simulate optimal positioning, an overwhelming majority were located in the 2nd and 3rd quadrants. These findings suggest that the articular cartilage disruption is measurable and varied among the lesser metatarsals. Moreover, the screw starting point and trajectory should be carefully selected to minimize articular disruption, especially in the smaller metatarsals.

Quantifying the impact of intramedullary screw placement through the metatarsal articular surfaces is a subject that has not been explored in the literature to our knowledge. Therefore, surface area measurements and analysis of trends in cartilage disruption could help inform the relationships between screw placement and potential cartilage damage. This question has been addressed in the context of upper extremity phalanx fractures looking at the quantifiable articular surface loss after screw insertion and the resulting range of motion loss of the proximal interphalangeal joint [8]. One study exploring this concluded that screw fixation resulted in minimal damage to the articular surface with an average surface loss of 4.4% across all digits [8]. Despite the percentage of cartilage disruption in our study being anywhere from 10 to 17%, the difference in the functionality and size of the phalanges in the hand and feet must be considered. Boe et al. assessed the impact articular surface defects from intramedullary screw fixation had on joint contact pressures [9]. An increase in joint contact pressure correlated with larger defects and further extension of the MCP. While this is not well-defined, the increase in joint contact pressures could be associated with the development of arthritis. A contact pressure greater than 15 N/mm2 is associated with chondrocyte death and extracellular matrix damage [9]. Their testing conditions included 50 N pressures that were equated to low impact physiologic stress [9]. Due to the low incidence of metatarsal arthritis, this concern is likely not as applicable in the foot but could be an area for further research. Notably, although post-traumatic arthritis and avascular necrosis are recognized concerns with intramedullary screw fixation, existing literature has not reported such complications following their use in metacarpals. Concerns regarding iatrogenic cartilage damage have been described in other areas of foot and ankle surgery. In a cadaveric Lisfranc injury model, Denove et al. demonstrated that transarticular fixation produces iatrogenic articular surface damage after simulated weightbearing [10]. This raised concerns regarding the risk of post-traumatic arthritis and contributed to the development of joint-sparing techniques. While this may reflect the limited duration of follow-up in current studies, it reinforces the notion that intramedullary screw fixation is generally well tolerated. Previous findings help bridge this gap by quantifying articular cartilage disruption in the metatarsals and emphasize the need for further studies [11]. Despite limited long-term follow-up, this supports the notion that screw fixation is generally well tolerated in the hand and supports the need for our results and further studies for use in the foot [12]. This could be due to shorter lengths of follow up in these studies, but the results are promising for reduction of immediate complications. This study also concluded similarly that smaller diameter screws resulted in less cartilage disruption and even with a 3.6 mm diameter screw the articular cartilage defect was 5% and not significant enough to outweigh the benefits [12]. 

There are several limitations to this study that should be recognized. Firstly, the analysis was conducted using a cadaveric model, which may not fully replicate in vivo conditions and leave us without the ability to comment on clinical outcomes. However, we were focused on the extent and location of cartilage disruption and are less likely to believe that this would significantly affect the function of the joint. Secondly, screw size was kept consistent across all specimens and metatarsals to avoid confounding with varied screw sizes. Clinically, some of the specimen metatarsals may accept a larger screw, and therefore we may underestimate the amount of cartilage damage that would be present in vivo. Additionally, all screws were placed in the middle third of the articular surface in line with the technique guide stating that a center starting point is ideal. This may not reflect the variability seen in actual surgical practice, as a starting point not centered in the metatarsal head could lead to different patterns of damage leading to instability or pain. The screws were also placed by a single foot and ankle surgeon who has experience utilizing these screws surgically for complex forefoot fractures. Keeping the medial to lateral axis consistent does improve the ability to draw conclusions about the trends of fixation on the plantar to dorsal axis and that the tendency was to put screws in the second or third quadrant despite the metatarsal size. The quadrant analysis, while useful for identifying common trajectories, may oversimplify the metatarsal anatomy and diversity of anatomy in patients. While the quadrant analysis is a simplification, it serves as a practical framework to quantify the spatial tendencies of screw placement that are often guided by experience and tactile feedback. Additionally, all measurements were obtained by a single observer and intraobserver reliability was not assessed. One final, but important limitation is that we do not comment on other structures that may be damaged such as the plantar plate or flexor tendons. While we did not assess for plantar plate disruption, we recognize that disrupting the plantar plate may lead to improper load distribution during the toe off phase of gait and over time may lead to instability of the metatarsophalangeal joint. Regarding the flexor tendon disruption, research was recently published characterizing the rate of flexor tendon disruption during insertion of intramedullary screw fixation of metatarsals [11]. 

Future research should explore the clinical context of these findings, including postoperative pain, range of motion and long-term risk of developing post-traumatic arthritis or malunion. Extended follow-up periods could provide insight into all possible complications and benefits of this technique. Advanced imaging could also be used to simulate joint loading of the foot and assess the biomechanical implications of various screw sizes and trajectories.

## Conclusions

This cadaveric study provides insights into the extent and location of articular cartilage disruption associated with retrograde intramedullary screw fixation of the lesser metatarsals. While cartilage injury is inevitable and an accepted consequence with this technique, the overall percentage of surface area affected ranges from approximately 10% in the second metatarsal to 17% in the fifth. Most screws were located in the second and third quadrants of the dorsal-to-plantar axis and all screws were in the middle third in the medial-to-lateral plane. This level of cartilage disruption carries unknown clinical consequences, and therefore further research is necessary to correlate these anatomic findings with long-term functional and radiographic outcomes.

## Data Availability

The data generated and/or analyzed during the current study are available from the corresponding author on reasonable request.
